# Synergism of Cattle and Bison Inoculum on Ruminal Fermentation and Select Bacterial Communities in an Artificial Rumen (Rusitec) Fed a Barley Straw Based Diet

**DOI:** 10.3389/fmicb.2016.02032

**Published:** 2016-12-15

**Authors:** Daniela B. Oss, Gabriel O. Ribeiro, Marcos I. Marcondes, WenZhu Yang, Karen A. Beauchemin, Robert J. Forster, Tim A. McAllister

**Affiliations:** ^1^Departamento de Zootecnia, Universidade Federal de ViçosaViçosa, Brazil; ^2^Coordenação de Aperfeiçoamento de Pessoal de Nível Superior, Ministério da EducaçãoBrasília, Brazil; ^3^Lethbridge Research and Development Centre, Agriculture and Agri-Food CanadaLethbridge, AB, Canada

**Keywords:** bacteria, barley straw, bison, *in vitro*, cattle, protozoa, rumen

## Abstract

This study evaluated the effect of increasing the proportion of bison relative to cattle inoculum on fermentation and microbial populations within an artificial rumen (Rusitec). The experiment was a completely randomized design with a factorial treatment structure (proportion cattle:bison inoculum; 0:100, 33:67, 67:33, and 100:0) replicated in two Rusitec apparatuses (*n* = 8 fermenters). The experiment was 15 d with 8 d of adaptation and 7 d of sampling. Fermenters were fed a diet of 70:30 barley straw:concentrate (DM basis). True digestibility of DM was determined after 48 h of incubation from d 13 to 15, and daily ammonia (NH_3_) and volatile fatty acid (VFA) production were measured on d 9–12. Protozoa counts were determined at d 9, 11, 13, and 15 and particle-associated bacteria (PAB) from d 13 to 15. Select bacterial populations in the PAB were measured using RT-qPCR. Fermenter was considered the experimental unit and day of sampling as a repeated measure. Increasing the proportion of bison inoculum resulted in a quadratic effect (*P* < 0.05) on straw, concentrate and total true DM disappearance and on straw and total neutral detergent fiber (aNDF) disappearance, with greater disappearances observed with mixed inoculum. There were no effect of source or proportion of inoculum on ADF disappearance (*P* > 0.05). Increasing bison inoculum linearly increased (*P* < 0.05) concentrate aNDF disappearance, total and concentrate N disappearance as well as total daily VFA and acetate production. A positive quadratic response (*P* < 0.05) was observed for daily NH_3_-N, propionate, butyrate, valerate, isovalerate and isobutyrate production, as well as the acetate:propionate ratio. Increasing the proportion of bison inoculum linearly increased (*P* < 0.05) total protozoa numbers. No effects were observed on pH, total gas and methane production, microbial N synthesis, or copies of 16S rRNA associated with total bacteria, *Selenomonas ruminantium* or *Prevotella bryantii*. Increasing bison inoculum had a quadratic effect (*P* < 0.05) on *Fibrobacter succinogenes*, and tended to linearly (*P* < 0.10) increase *Ruminococcus flavefaciens* and decrease (*P* < 0.05) *Ruminococcus albus* copy numbers. In conclusion, bison inoculum increased the degradation of feed protein and fiber. A mixture of cattle and bison rumen inoculum acted synergistically, increasing the DM and aNDF disappearance of barley straw.

## Introduction

Animal agriculture must find alternative and cost-effective feed ingredients to remain profitable in a projected future of increased food demand and costs (Thornton, 2010). Lignocellulosic crop residues could fulfill this need if their digestion could be optimized. Ruminants are unique in their ability to utilize lignocellulosic feeds as the rumen microbial consortia is considered to be one of the most efficient microbial systems at degrading lignocellulosic biomass (Flint et al., 2008). However, even in light of this efficiency, often less than 50% of carbohydrates in low quality forages such as straw are digested by cattle (Sari et al., 2015). Comparing the rumen microbial community of ruminants that are more effective at degrading lignocellulose to those that are less efficient may expand our understanding of the key microbes and their roles in plant cell wall deconstruction.

The American buffalo or bison (*Bison bison*) and the domestic bovine (*Bos taurus*) are ruminant species that evolved in different environments (Koch et al., 1995), a factor that may account for the tendency of bison to graze lower quality forages than cattle (Peden et al., 1974). Some studies have suggested that bison are more efficient than cattle at digesting poor-quality forages (Richmond et al., 1977; Hawley et al., 1981a,b). Proposed mechanisms for this heightened efficiency include a reduction in the rumen particulate passage rate, an increase in nitrogen (N) recycling, as well as differences in ruminal microbial populations (Hawley et al., 1981b). However, relatively little is known about ruminal fermentation characteristics and microbial populations in bison. Compared to cattle, bison possess a greater percentage of *Fibrobacter succninogenes, Ruminococcus albus*, and *Ruminococcus flavefaciens* in rumen contents (Varel and Dehority, 1989). Higher ruminal ammonia (NH_3_) concentrations and total protozoal numbers, and a differing species density (greater *Dasytricha* spp., *Eudiplodinium maggii, Eudiplodinium bursa*, and *Epidinium* spp.) was also observed for bison compared to cattle when both were fed poor-quality hay (Towne et al., 1988).

The manipulation of the ruminal microbial community to improve fiber digestion has been largely unsuccessful (Weimer, 2015). In a classic study, despite massive inoculation of highly efficient cellulolytic bacteria strains to nearly empty rumens, the inoculated bacteria failed to colonize the rumen and were washed out within 24 h (Varel et al., 1995). There is evidence suggesting that the rumen microbiome may be host-specific, possibly raising barriers to the establishment of introduced microbes across different hosts (Weimer et al., 2010). A possible reason for this is that each individual animal possess a microbial community that is able to reconstitute itself even after serious perturbation, reflecting the ecological principles of inertia and resilience (Westman, 1978). The alternative use of the *in vitro* semi-continuous rumen simulation system (Rusitec) allows testing the effect of different rumen inoculums (i.e., cattle vs. bison) on fiber digestion under more standardized environmental conditions (i.e., temperature, pH, passage rate) as a step toward defining the importance of host specificity.

Therefore, we hypothesized that ruminal inoculum from bison would promote greater degradation of lignocellulose in the Rusitec as compared to ruminal inoculum from cattle. Thus, the objective of this study was to evaluate the effect of increasing the proportion of bison rumen inoculum on fermentation parameters, microbial populations and the digestion of barley straw using the Rusitec.

## Materials and methods

The present experiment was conducted at the Agriculture and Agri-Food Canada Research and Development Centre in Lethbridge (LRDC), Alberta, Canada. Donor animals used in the experiment were cared for in accordance with the guidelines of the (Canadian Council on Animal Care, 2009) and protocols were approved by the Lethbridge Research and Development Centre Animal Care Committee.

### Experimental design and treatments

The experiment was a completely randomized design with four treatments (ruminal inoculum) carried out in 16 Rusitec fermenters (*n* = 4/treatment) as described by Czerkawski and Breckenridge (1977). The duration of the experiment was 15 d. The Rusitecs were allowed to reach steady state over the first 8 d, followed by a 7 d sampling period (d 9 to 15). Treatments consisted of increasing replacement of ruminal inoculum from cattle (*Bos taurus*) with contents from bison (*Bison bison*) with the following proportions of cattle:bison inoculum in the fermenters: (1) 100:0; (2) 67:33; (3) 33:67; and (4) 0:100. Rusitecs were fed a diet of barley straw (700 g/kg DM) and concentrate mix (300 g/kg DM basis; 667 g/kg of corn distiller's dried grains with solubles (DDGS), 266 g/kg of canola meal, 57 g/kg of mineral/vitamin supplement and 10 g/kg of urea; Table 1). Feeds were ground through a 4-mm screen (Arthur H. Thomas Co., Philadelphia, PA, USA), with the straw and concentrate placed in separate polyester bags (for concentrate: 50 × 100 mm; pore size = 50 μm; for straw: 100 × 200 mm; pore size = 50 μm; ANKOM; Ankom Technology Corp.).

**Table 1:** **Chemical composition of barley straw and concentrate used as diet**.

**Item**	**Ingredients**		**Diet**
	**Barley straw^a^**	**Concentrate^b^**	
DM, g/kg	922	898	915
OM, g/kg DM	915	896	909
N, g/kg DM	9	56	23
CP, g/kg DM	58	352	146
aNDF, g/kg DM	729	309	603
ADF, g/kg DM	473	131	370

a *70% on DM basis*.

b *30% on DM basis; 66.7% of DDGS, 26.6% of canola meal, 5.7% of supplement and 1% of urea*.

### Experimental apparatuses and incubations

Each Rusitec apparatus was equipped with eight 920 mL fermenters. Each fermenter had an inlet for the infusion of buffer and an out port for the collection of effluent. Fermenters were immersed in a water-bath maintained at 39°C. The four treatments were randomly assigned to duplicate fermenters within each Rusitec apparatus (four replications per treatment). The experiment was started by filling each fermentation vessel with 180 mL of warmed McDougall's buffer (McDougall, 1948) modified to contain 0.3 g/L of (NH_4_)_2_SO_4_ and 720 mL of strained rumen fluid as described by Ribeiro et al. (2015).

Cattle inoculum was obtained before feeding from a pool of 16 ruminally cannulated heifers fed the same diet as the Rusitec. Bison inoculum was obtained from a pool of 32 bison rumens collected at a local slaughterhouse. The bison were fed a forage diet containing barley silage and oats (75:25 DM basis). The time from slaughter of the bison to initiation of fermentation in the Rusitec was less than 2 h. Rumen contents from the bison were pooled in a large heated vessel, a representative sample of fluid was collected and filtered through four layers of cheesecloth into an insulated thermos and immediately transported to the laboratory. Approximately 400 g (200 g from cattle, 200 g from bison) of ruminal solid digesta was also collected for the initial inoculation of the fermenters. All fermenters were inoculated at the same time and a large number of each ruminant species was deliberately sampled to ensure that the sample collected represented the core microbiome of the herd as opposed to an individual.

On day 0, a solid digesta bag (1 bag, 20 g wet wt) according to inoculum proportions for each treatment, along with two additional bags containing either barley straw (7 g DM) or concentrate (3 g DM) were included in each fermenter. After 24 h (day 1), the solid rumen digesta was replaced with two bags, one containing straw, and another containing concentrate. From day 1 onwards, fermenters now containing four bags were opened daily to replace the two bags that had been incubated for 48 h. Artificial saliva was continuously infused into the fermenters at a dilution rate of 2.9%/h. During bag exchange, each fermentation vessel was flushed with O_2_-free CO_2_ to maintain anaerobic conditions. Effluent was collected in a 2.0 L Erlenmeyer flask and measured daily during feed bag exchange.

### Measurements

#### True DM disappearance

True DM disappearance at 48 h was determined from day 13 to 15. Feed bags (forage and concentrate) were removed from each fermenter, processed together in a paddle blender (Stomacher 400; Seward Ltda, Worthing, UK) to obtain feed particle-associated (FPA) and feed particle-bound (FPB) bacterial fractions, and dried at 55°C for 48 h. Upon removal from fermenters, both straw and concentrate bags were gently squeezed to expel excess liquid. Bags were placed together in a plastic bag with 20 mL of buffer (McDougall, 1948) and processed in the paddle blender for 60 s at 230 RPM. The processed liquid was exuded, poured off and retained. Feed residues were then washed twice with 10 mL of McDougall's buffer in each wash. The wash buffer was retained and pooled with the fluid obtained after paddle blending to obtain the FPA bacterial fraction, and total volume was recorded. Bacteria attached to the washed solid feed residues were considered to represent the FPB bacterial fraction. The true DM disappearance was calculated by the difference between feed initially added to the bag and feed residues minus FPB (i.e., minus microbial mass).

#### Fermentation

Fermentation gas was collected daily into reusable 2 L gas-tight vinyl collection bags (Curity®; Conviden Ltd., Mansfield, MA, USA) that were attached to each of the effluent vessels. Gas bags were clamped before opening the fermenters or effluent collection. Just before feed bag exchange, daily total gas production (d 1 to d 15) from each fermenter was measured using a gas meter (Model DM3A, Alexander-Wright, London, England, UK). From d 9 to d 15, before total gas measurements, gas samples (20 mL) were collected from the septum of the collection bags using a twenty-six-gauge needle (Becton Dickinson, Franklin Lakes, NJ, USA) and transferred to evacuated 6.8 mL Exetainer vials (Labco Ltd., Wycombe, Bucks, UK) for CH_4_ analysis. The CH_4_ concentration in gas samples was determined as described by Avila-Stagno et al. (2014) using a Varian gas chromatograph equipped with GS-Carbon-PLOT 30 m × 0.32 mm × 3 μm column and thermal conductivity detector (Agilent Technologies Canada Inc., Mississauga, ON, Canada).

The pH of the fluid from each fermenter was recorded (Orion model 260A, Fisher Scientific, Ottawa, ON, Canada) daily (d 1 to d 15) at the time of feed bag exchange. The amount of effluent produced daily was measured with a graduated cylinder. To determine VFA concentration in fermenter effluent, subsamples (2.5 mL) were collected directly from the effluent flasks containing 20 mL of 3.66 *M* H_2_SO_4_ (20%, vol/vol) (Giraldo et al., 2007) at the time of feed bag exchange. Samples were placed in screw-cap vials, preserved with 500 μL of 25% (w/w) metaphosphoric acid, and immediately frozen at −20°C until analyzed. At the same time, 2.5 mL subsamples of effluent were also collected, placed in screw-cap vials and preserved with 500 μL of H_2_SO_4_ (1%, vol/vol) for determination of NH_3_-N. The concentrations of VFA and NH_3_-N (mmol/L) were multiplied by daily effluent production (L/d) to determine VFA and NH_3_-N production (mmol/d).

#### Protozoa

Protozoa were enumerated on d 3, 9, 11, 13, and 15 from each fermenter. Bags were gently pressed to expel fermentation fluid and a 2.5 mL subsample of rumen fluid was obtained and preserved using 2.5 mL of methyl green formalin-saline solution (Ogimoto and Imai, 1981). Protozoa samples were stored in the dark at room temperature until enumerated by light microscopy using a Levy–Hausser counting chamber (Hausser Scientific, Horsham, PA, USA).

#### Microbial protein synthesis

Bacteria in the fermenters were labeled using ^15^N. On day 7, 0.3 g/L (NH_4_)_2_SO_4_ in McDougall's buffer was replaced with 0.3 g/L ^15^N-enriched (NH_4_)_2_SO_4_ (Sigma Chemical Co., St. Louis, MO, USA; minimum ^15^N enrichment 10.01 atom%) until the end of the experiment. On d 13, 14, and 15, daily effluent samples were preserved with 3 mL of a sodium azide solution (20%; wt/vol) and 40 mL were subsampled for isolation of liquid-associated bacteria.

To determine ^15^N concentration, effluent liquid samples were centrifuged (20,000 × g, 30 min, 4°C) and the resulting pellets were washed using de-ionized water and centrifuged 3 times (20,000 × g, 30 min, 4°C). The pellets were then re-suspended in distilled water and frozen at −20°C until lyophilized. The FPA bacterial samples collected after stomaching were centrifuged (500 × g, 10 min, 4°C) to remove large feed particles and the supernatant was decanted and centrifuged (20,000 × g, 30 min, 4°C) to isolate a bacterial pellet which was washed 3 times as previously described. The pellet was then resuspended in distilled water and stored at −20°C. Washed feed residues (FPB fraction) were dried at 55°C for 48 h, weighed for DM determination, ball ground (MM 400; Retsch Inc., Newtown, PA, USA), and analyzed for total N and ^15^N by combustion analysis using a mass spectrometer (NA 1500, Carlo Erba Instruments).

#### Quantification of microbial 16S rRNA marker genes by qPCR

Samples prepared from FPA were also used to estimate density of selected bacterial populations using real-time polymerase chain reaction (qPCR) (Narvaez et al., 2013). The 48-h incubated residues from d 2, 13, 14, and 15 were processed as described above to obtain the FPA and to quantify microbial populations before (d 2) and after adaptation (d 13, 14, and 15).

Total DNA was extracted from FPA samples using a Qiagen QIAmp DNA Stool Mini Kit (Qiagen Inc., Valencia, CA, USA) according to the manufacturer's instructions, using a slight modification to enhance the yield of DNA from Gram-positive bacteria. Briefly, approximately 30 mg of each sample was suspended in 1.4 mL of ASL buffer (Stool lysis buffer; Qiagen Inc., Valencia, CA, USA) with 0.4 g of sterile zirconia beads (0.3 g of 0.1 mm and 0.1 g of 0.5 mm), and homogenized for 3 min at maximum speed (30/s) using a Qiagen Tissue Lyser II (Qiagen Inc., Valencia, CA, USA). The suspension was heated at 95°C and mixed gently in a thermomixer (Eppendorf-Thermomixer comfort, Eppendorf Ltd, Mississauga, ON, Canada) for 5 min before processing according to the manufacturer's protocol. Total DNA was eluted in 200 μL of Buffer AE (Elution buffer; Qiagen Inc., Valencia, CA, USA) and quantified using a Quant-iT™PicoGreen® dsDNA Assay Kit (Invitrogen Canada Inc., Burlington, ON, Canada) with a NanoDrop 3300 fluorometer (Thermo Scientific, Wilmington, DE, USA). Real-time PCR was used to quantify 16S rRNA copies for total bacteria and sequences specific to *Fibrobacter succinogenes* subsp. S85, *Prevotella bryantii* B_1_4, *Ruminococcus albus* 7, *Ruminococcus flavefaciens* C94 and *Selenomonas ruminantium* subsp. Lactilytica (ATCC 19 205). Primers and annealing temperatures were as described previously for *F. succinogenes, P. bryantii, R. flavefaciens, S. ruminantium* (Tajima et al., 2001) and *R. albus* (Wang et al., 1997). Detailed information on PCR cycling conditions, plasmid standards and reference bacterial strains are as described by Wang et al. (2009). Universal 16S primer sequences: 16S-F: CTCCTACGGGAGGCAGCAGT and 16S-R: TTACCGCGGCTGCTGGCAC were used to detect total bacteria using amplification conditions: one cycle at 95°C for 3 min, 30 cycles of denaturation at 95°C for 15 s, annealing at 60°C for 30 s, and extension at 72°C for 30 s. A total of 25 μL of PCR mixture containing 150 nM of each 16S primer was used. For PCR, all samples were normalized to contain 40 ng/μL and 2 μL of extracted DNA template per reaction.

#### Chemical analysis

Forage and concentrate bag residues were dried at 55°C for 48 h and pooled over 3 days (d 9, 10 and 11, and d 13, 14, and 15 for each fermenter) to ensure that there was sufficient sample for chemical analysis. Samples were ground through a 1 mm screen in a Wiley mill (standard model 4; Arthur H. Thomas Co., Philadelphia, PA, USA) before chemical analysis. Substrates were analyzed for DM (method no. 930.15) and ash content (method no. 942.05) according to AOAC (2006). The concentration of neutral detergent fiber (aNDF) was assayed with a heat stable amylase and sodium sulphite and expressed inclusive of residual ash (Van Soest et al., 1991). The concentration of acid detergent fiber (ADF) was determined according to method 973.18 (AOAC, 2006). The concentration of total N (method no. 990.03; AOAC, 2006) was determined by combustion analysis using a mass spectrometer (NA 1500, Carlo Erba Instruments). Concentrations of VFA and NH_3_-N in the liquid effluent were analyzed by gas chromatography (Wang et al., 2001) and the modified Berthelot method (Rhine et al., 1998), respectively.

### Calculations

Disappearance of aNDF, ADF and N was determined by the difference between the amount of those components in the substrates before and after incubation. Total disappearances (forage + concentrate) were calculated by difference between the amount substrate (forage + concentrate) incubated and the residue after incubation (forage + concentrate).

Total daily effluent microbial N (MN) was calculated using the N concentration (%) determined for the microbial pellet (harvested from the 40 mL effluent subsample) multiplied by the microbial weight in the total effluent (mg/day). Microbial weight in the total effluent was calculated by multiplying daily effluent production (mL) by the microbial density (mg/mL), which was determined in a 40 mL subsample. The microbial N production (mg/day) from the FPA fraction was calculated by multiplying the N concentration (%) in the FPA microbial pellet by the microbial weight of the FPA fraction (mg/day). The MN production (mg/day) from the FPB fraction was estimated using the following equation:

$$\begin{array}{l} \text{MN}=\frac{\text{APE in RN}}{\text{APE in MN}}\times \text{RN} \end{array}$$

where atom per cent excess (APE) in residue nitrogen (RN) is the percent excess of ^15^N in solid residue, APE in MN is the percent excess of ^15^N in the microbial N fraction of the FPA and RN is the total N (mg) in the residue. Total daily MN production (mg/day) was calculated as the sum of microbial production in the effluent, FPA, FPB of straw residues and FPB of concentrate residues (Ribeiro et al., 2015).

True DM disappearance was calculated by subtracting the microbial mass from feed residues. Microbial mass in feed residues was calculated by multiplying MN production (mg) in feed residues by the microbial mass per mg of MN (g of DM of microbial pellet/mg of MN). Microbial mass per mg of MN was determined in FPA bacterial pellets. Ammonia-N and daily VFA production were calculated by multiplying the concentration of the fermentation end product in the effluent by the daily production of effluent. The total bacterial and specific bacteria *16S rRNA* used an absolute quantification method with comparison to cloned standards of known quantity as described (Wang et al., 1997). Copies of *16S rRNA* were analyzed individually for the five bacterial species and expressed as a percentage of total bacterial *16S rRNA* present in the FPA fraction.

### Statistical analysis

Data were analyzed using the MIXED procedure of SAS (SAS Inc., Cary, NC, USA). The model included the fixed effects of inoculum, day and inoculum × day interactions with the day of sampling from each fermenter treated as a repeated measure with individual fermenter considered as the experimental unit. The minimum values of Akaike's Information Criterion was used to select the covariance structure among compound symmetry, heterogeneous compound symmetry, autoregressive, heterogeneous autoregressive, Toeplitz, unstructured and banded for each parameter. The effect of day and its interactions were removed from the model when we had just 1 day of sampling or when different day samples were combined for analysis. Outliers were identified and excluded from the dataset when the studentized residual was outside the range of −2.5 to 2.5 (Neter et al., 1996; Kaps and Lamberson, 2004). Orthogonal polynomial contrasts were performed to determine if replacing cattle inoculum with increasing concentrations (0, 33, 67, and 100%) of bison inoculum resulted in a linear or quadratic effect on measured parameters. Significance was declared at *P* ≤ 0.05, and trend was discussed when 0.05 < *P* < 0.10.

## Results

### Dry matter and nutrient disappearance and fermentation characteristics

Increasing the proportion of bison inoculum resulted in a positive quadratic effect (*P* < 0.05) on total (straw + concentrate), straw and concentrate true DM disappearance, and on straw and total aNDF disappearance (Table 2). For disappearance of straw aNDF, five data points from a total of 32 were identified as having studentized residual outside the range of −2.5 to 2.5 and were removed before contrast analysis. Concentrate aNDF disappearance increased linearly (*P* = 0.008) with increasing proportions of bison inoculum. The relative proportions of cattle:bison rumen inoculum did not affect (*P* > 0.22) total or strawADF disappearance, but concentrate ADF disappearance increased linearly with increasing proportions of bison inoculum (*P* = 0.02). Increasing the proportion of inoculum from bison also resulted in a linear increase in total (*P* = 0.01) and concentrate (*P* = 0.04) N disappearance, but strawN disappearance was unaffected.

**Table 2:** **Effect of differing proportions of cattle and bison rumen inoculum on true DM, aNDF, ADF, and nitrogen (N) disappearance (g/kg) of a barley straw diet in the rumen simulation technique (Rusitec)**.

**Item**	**Cattle:Bison Inoculum**				**SEM**	**Linear**	**Quadratic**
	**100:0**	**67:33**	**33:67**	**0:100**			
**TRUE DM DISAPPEARANCE**^a^							
Total	503	520	528	503	5.2	0.73	0.002
Barley straw	401	408	419	391	5.6	0.44	0.01
Concentrate	747	787	790	771	11.1	0.15	0.02
**aNDF DISAPPEARANCE**^b^							
Total	360	382	381	367	6.0	0.54	0.003
Barley straw	326	330	337	316	5.1	0.37	0.02
Concentrate	566	639	653	652	2.3	0.01	0.09
**ADF DISAPPEARANCE**^b^							
Total	328	332	329	323	7.7	0.60	0.47
Barley straw	310	310	307	297	7.4	0.21	0.52
Concentrate	492	533	545	545	17.0	0.02	0.22
**N DISAPPEARANCE**^b^							
Total	617	695	692	697	21.7	0.01	0.08
Barley straw	388	400	417	398	19.8	0.59	0.43
Concentrate	725	811	826	815	31.6	0.04	0.11

a *Samples from days 13 to 15*.

b *Samples from days 9 to 15*.

The source of rumen inoculum did not affect (*P* ≥ 0.10) pH, total gas production, methane production, or microbial N production (total, FPB, FPA or Effluent; Table 3). Increasing the proportion of inoculum from bison resulted in a linear increase in total daily VFA (*P* = 0.04) and acetate (*P* = 0.03) production. Increasing levels of bison inoculum had a quadratic effect (*P* < 0.05) on daily NH_3_-N, propionate, butyrate, valerate, isovalerate and isobutyrate production and acetate:propionate ratio (C2:C3). Increasing the proportion of inoculum from bison also resulted in a linear increase in numbers (*P* < 0.001) of total protozoa on d 3, and after adaptation on d 9, 11, 13, and 15 (*P* < 0.001).

**Table 3:** **Effect of differing proportions of cattle and bison rumen inoculum on pH, ammonia-N, volatile fatty acid (VFA), total gas, methane (CH_**4**_), microbial N production and protozoa for a barley straw diet using the rumen simulation technique (Rusitec)**.

**Item**	**Cattle:Bison Inoculum**				**SEM**	**Linear**	**Quadratic**
	**100:0**	**67:33**	**33:67**	**0:100**			
pH^a^	6.80	6.77	6.80	6.80	0.015	0.96	0.32
Ammonia-N, mmol/d^b^	6.35	7.46	7.53	7.65	0.163	<0.001	0.007
Total VFA, mmol/d^b^	37.1	38.9	39.1	40.1	0.90	0.04	0.63
Acetate (C2), mmol/d^b^	23.5	24.4	24.8	26.1	0.78	0.03	0.86
Propionate (C3), mmol/d^b^	10.0	10.6	10.3	9.7	0.17	0.12	<0.001
Butyrate, mmol/d^b^	2.3	2.3	2.4	2.7	0.04	<0.001	<0.001
Valerate, mmol/d^b^	0.59	0.69	0.66	0.67	0.011	0.001	<0.001
Isovalerate, mmol/d^b^	0.46	0.60	0.60	0.61	0.022	<0.001	0.003
Isobutyrate, mmol/d^b^	0.30	0.36	0.35	0.37	0.011	<0.001	0.04
C2:C3^b^^,^ ^c^	2.36	2.31	2.42	2.70	0.048	<0.001	<0.001
Gas, L^a^	1.55	1.46	1.39	1.39	0.134	0.36	0.74
CH_4_, mg/d^a^	37.9	37.3	37.2	37.8	4.63	0.97	0.93
CH_4_, mg/g incubated DM^a^	4.1	4.1	4.1	4.1	0.51	0.97	0.93
CH_4_, mg/g degraded DM^a^	8.0	7.6	7.6	7.8	1.03	0.88	0.80
Production of microbial N, mg/d^d^							
Total	55.9	56.5	57.6	56.3	1.48	0.73	0.54
FPB^e^ Concentrate	2.1	2.2	1.7	2.2	0.29	0.91	0.48
FPB Barley straw	20.0	17.4	17.8	17.7	1.23	0.26	0.33
FPA^f^	5.6	5.4	5.6	5.5	0.26	0.82	0.97
Effluent	30.6	31.4	32.5	30.9	1.03	0.64	0.23
Protozoa (× 10^3^/mL), d 3	16.0	25.5	59.0	71.0	9.10	<0.001	0.89
Protozoa (× 10^3^/mL)^g^	7.2	7.9	14.9	17.0	1.83	<0.001	0.70

a *Samples from days 9 to 15*.

b *Samples from days 9 to 12*.

c *Acetate: propionate ratio*.

d *Samples from days 13 to 15*.

e *Feed particle-bound*.

f *Feed particle-associated*.

g *Samples from days 9, 11, 13 and 15*.

### Microbial population

Prior to adaptation, qPCR analysis revealed a linear decrease in 16S rRNA copies of total bacteria (*P* = 0.03) and in the percentage of *F. succinogenes (P* = 0.004) with increasing proportions of inoculum from bison (Table 4). A positive quadratic effect was observed for the percentage of *R. albus* (*P* = 0.05) whereas increasing the proportion of inoculum from bison did not affect the percentage of *S. ruminantium* or *P. bryantii*.

**Table 4:** **Effect of differing proportions of cattle and bison rumen inoculum on total ***16S rRNA*** bacterial gene copies and on relative abundance of marker genes for specific bacterial species (% of total bacterial ***16S rRNA***) in the feed particle-associated (FPA) fraction of barley straw diet, before and after adaptation in the rumen simulation technique (Rusitec)**.

**Item**	**Cattle:Bison Inoculum**				**SEM**	**Linear**	**Quadratic**
	**100:0**	**67:33**	**33:67**	**0:100**			
**BEFORE ADAPTATION (d 2)**							
Total bacterial 16S rRNA copies^a^ (× 10^10^)	75.4	72.3	49.8	48.8	9.09	0.03	0.91
**SPECIFIC MICROBIAL POPULATION (% × 10^−1^)**							
*F. succinogenes*	4.22	2.54	0.87	0.34	0.888	0.004	0.49
*R. flavefaciens*	0.40	0.55	0.58	0.38	0.098	0.99	0.10
*R. albus*	1.09	1.24	1.79	1.07	0.197	0.57	0.05
*S. ruminantium*	4.68	1.81	5.17	2.17	0.516	0.11	0.90
*P. bryantii*	0.52	2.40	3.04	1.70	1.259	0.44	0.18
**AFTER ADAPTATION (d 13, 14, 15)**							
Total bacterial 16S rRNA copies^a^ (× 10^10^)	48.4	41.4	47.4	49.6	3.114	0.49	0.16
**SPECIFIC MICROBIAL POPULATION (% × 10^−1^)**							
*F. succinogenes*	4.59	6.29	5.93	3.51	0.992	0.42	0.05
*R. flavefaciens*	0.31	0.34	0.30	0.43	0.033	0.09	0.16
*R. albus*	0.46	0.35	0.34	0.35	0.032	0.03	0.10
*S. ruminantium*	0.34	0.18	0.32	0.25	0.086	0.75	0.63
*P. bryantii*	0.12	0.04	0.11	0.03	0.044	0.38	0.99

a *Total bacterial 16S rRNA gene copies in FPA were the total amount estimated from daily residual outputs of each fermentation vessel*.

After adaptation, the source of inoculum did not affect *16S rRNA* copies of total bacteria or the percentages of *S. ruminantium* or *P. bryantii* in FPA fractions (Table 4). However, increasing the proportion of bison inoculum had a positive quadratic effect on *F. succinogenes* (*P* = 0.05), a linear decrease on *R. albus* (*P* = 0.03), and a tendency of a linear increase on *R. flavefaciens* (*P* = 0.09) proportion.

## Discussion

In the present study the bison used as rumen inoculum donors were fed a diet that differed from that of cattle, as we were unable to convince commercial producers to feed bison an extremely low quality straw diet. However, bison were fed a forage based diet consisting of barley silage and oats (75:25 DM basis), but even with this difference solid ruminal digesta from bison possessed similar crude protein, NDF and ADF concentrations to that of cattle. Fermenters containing inoculum from both host species were allowed to adapt to the concentrate—straw diet for a period of 8 d, a period that is 6 d longer than the 48 h that have been recently reported to be sufficient to allow microbial populations in the Rusitec to stabilize (Lengowski et al., 2016). Although, there is a possibility that diet may have influenced the nature of the microbial populations within the initial inocula, if it was an overriding factor one would predict that the aNDF disappearance in fermenters inoculated with digesta from cattle should be far higher than those inoculated with bison. The fact that the aNDF disappearance was highest in fermenters containing a mixture of cattle and bison contents suggests that some of this synergy arose from the mixing of microbiomes from different ruminant hosts.

### DM and nutrient disappearance and fermentation characteristics

Bison inoculum alone did not improve total DM or ADF disappearance compared to cattle inoculum. However, mixtures of bison and cattle rumen inoculum improved straw and total DM and aNDF disappearances as compared to either bison or cattle rumen inoculum alone. These results suggest possible synergistic effects of the mixture of cattle and bison inoculum on DM and aNDF degradation of straw based diets. Synergy has been previously reported in pure cultures when a non-fibrolytic *Prevotella ruminicola* strain was co-cultured with either *F. succinogenes* or *R. flavefaciens*, as fiber digestion was improved compared to that of the fibrolytic species alone (Osborne and Dehority, 1989; Fondevila and Dehority, 1994). Also, *F. succinogenes* acted synergistically with the fungal species, *Caecomyces*, to degrade perennial ryegrass (*Lolium perenne*) stem fragments, suggesting that these prokaryotic and eukaryotic species may have complementary fibrolytic activities (Joblin et al., 2002). Although synergies among ruminal microorganisms within consortia from different ruminant species are likely far more complex, these laboratory studies do demonstrate that such synergies do occur.

Corn DDGS and canola meal, the main concentrate ingredients in this study, are common protein supplements fed to cattle and both have high proportion of rumen-undegradable protein (RUP; Santos et al., 1998). In spite of this fact, N disappearance from the concentrate increased with increasing proportion of bison inoculum. As the urea portion of the concentrate can be considered completely degraded or washed out of the fermenters for all treatments, the increased N disappearance is most likely a result of a specific effect of the bison inoculum on RUP. Furthermore, the increase in N disappearance from the concentrate corresponded to an increase in NH_3_-N. High ruminal NH_3_-N concentration has been associated with large numbers of protozoa in rumen contents (Veira et al., 1983; Towne et al., 1988), presumably a reflection of the predatory activity of these eukaryotes against bacteria (Belanche et al., 2011, 2012) and their ability to degrade feed protein (Ushida et al., 1986). Previous studies demonstrated greater feed protein degradation of diets with low protein solubility in faunated ruminants compared to protozoa-free ruminants (Ushida and Jouany, 1985, 1986; Ushida et al., 1986). The present study is in agreement with these findings, as the linear increase in total and concentrate N disappearance and daily NH_3_-N production observed with increasing proportions of bison inoculum is consistent with the increase in total protozoa numbers.

The greater number of protozoa in the Rusitec with increasing proportion of bison inoculum likely reflects the greater protozoa numbers in the rumen of bison than cattle *in vivo*. Greater total protozoa numbers in the rumen of bison compared to cattle have been previously reported when both were fed similar diets (Towne et al., 1988).

The linear increase in total VFA and the quadratic increase in valerate, isovalerate and isobutyrate production observed with increasing proportions of bison inoculum is likely a reflection of the linear increase in CP degradation (N disappearance) and the catabolism of branched-chain amino acids (Lindsay and Reynolds, 2005). Lack of effect on total gas and CH_4_ production is somewhat surprising given the observed linear or quadratic changes in DM and CP degradation and the differences in C2:C3 ratio. The observed increase in C2:C3 ratio with increasing proportion of ruminal inoculum from bison was expected to correspond to an increase in CH_4_ production (Moss et al., 2000), particularly because production of microbial N (total, FPB concentrate or forage, FPA and effluent) was not affected as microbial synthesis can serve as a hydrogen sink. However, the lack of an effect on microbial N is consistent with the similar total copies of bacterial 16S rRNA observed across treatments after fermenters were adapted.

Some studies have suggested that bison have a superior ability to digest low-quality forages as compared to cattle (Peden et al., 1974; Richmond et al., 1977; Hawley et al., 1981a), but no studies have compared possible synergy between the two sources of inoculum. Hawley et al. (1981b) observed that the *in vivo* digestion coefficients of DM, CP, aNDF and ADF from sedge hay were greater in bison than in Hereford steers. They suggested that digestibility of DM, CP and fiber is superior in bison when poor-quality, low-protein diets are fed. Richmond et al. (1977) also observed in an *in vivo* experiment, greater digestibility of sedge and grass hays (7–8% CP) in bison than cattle, but the digestibility of alfalfa (19% CP) was similar between these two species. These authors theorized that the superior ability of bison to digest poor-quality, low protein forages as compared to cattle, was due to their greater ability to recycle ruminal N, a reduced rate of ruminal particulate passage and differences in ruminal microbial populations. These first two factors would not be responsible for differences in digestion in the artificial rumen as feed bags were retained in fermenters for the same period of time, and recycling of N did not occur.

### Microbial population

The reduction in total copies of bacterial 16S rRNA observed with increasing bison rumen inoculum at the beginning of the Rusitec study (d 2), with a lack of a difference after adaptation, may reflect the fact that cattle were more adapted to the diet fed to the fermenter, whereas the bison were not. Another possibility is that there were carryover effects of the differing composition of the diets fed to the donor animals that influenced initial bacterial populations that were introduced into the Rusitec. Alternatively, it might indicate that the longer delay between obtaining the inoculum and initiating the fermenters for bison compared with cattle resulted in lower bacterial populations. The lack of differences in total copies of bacterial 16S rRNA in fermenters is a reflection that adaptation occurred and in part likely reflects that the dilution rate and substrates provided in all fermenters was identical.

The quadratic effect observed for *F. succinogenes* after adaptation is consistent with the quadratic response observed for total aNDF and DM disappearance. This result indicates that *F. succinogenes* was positively affected by cattle and bison rumen inoculum mixture. It is possible that the disturbance in the microbial community created by the mixture of rumen inoculum from the different species created favorable conditions for the growth of *F. succinogenes*.

The interactions among major fibrolytic species in the rumen (*F. succinogenes, R. flavefaciens* and *R. albus*) are complex, and some studies have documented antagonistic responses in cellulose digestion among these species *in vitro* (Odenyo et al., 1994; Shi et al., 1997). Our results are suggestive of this antagonistic behavior as we observed a linear decrease in the *R. albus* population, and a tendency for a linear increase in *R. flavefaciens* and a quadratic effect on *F. succinogenes* population with increasing proportions of bison inoculum. There is evidence that *R. albus* and *R. flavefaciens* are capable of producing inhibitors (bacteriocin-like factors) that suppress the growth of *R. flavefaciens* and *F. succinogenes*, respectively (Chen and Weimer, 2001).

The Rusitec eliminates any potential differences between cattle and bison in chewing efficiency, passage rate, N recycling, and nutrient absorption, and thus enables a comparison of the inoculum alone. According to a recent global rumen census (Henderson et al., 2015), differences in the rumen microbial community are predominantly attributable to diet, with the host being less influential. A recent study by Witzig et al. (2015) evaluated the effect of different donor animal species (cattle vs. sheep) and diet fed to the donor animals (hay-concentrate vs silage-based diets) on the microbial community established in the Rusitec. Their results suggest that the donor animal diet had a substantial impact on composition of the microbial community within the Rusitec and that the effect of the donor animal species itself was of minor consequence. In our study, bison and cattle were fed different diets, but of similar aNDF content and most measurements were made after adaptation. Thus, composition of the diet fed to host animals may have influenced fermentation immediately after the Rusitec started, but diet was likely not a factor once the system was adapted. We recognize that the Rusitec system is a simplified microbiome reflecting the starting cultures and that microbial interactions may be more complex *in vivo*, but nevertheless the technique may aide in the identification of strategies that improve digestion efficiency.

## Conclusions

To our knowledge, this is the first *in vitro* continuous culture experiment examining cross species inoculation of fermenters. Overall, bison inoculum more readily degraded feed protein than cattle inoculum, with a mixture of inoculums synergistically increasing the DM and aNDF disappearance of a barley straw based diet. Ruminal inoculum from bison alone did not promote greater fiber digestion as compared to that obtained from cattle. Rumen inoculation across ruminant species may be a means of increasing ruminal fiber and protein degradation, but this still needs to be investigated *in vivo*. In addition, microbiome studies are needed to explore whether the higher digestibility observed in mixed inocula correlates with greater bacterial or protozoal biodiversity.

## Author contributions

DO, GR, and TM designed the experiment; DO and GR conducted the research; GR, KB, and RF analyzed the data and DO, GR, TM, KB, and MM wrote the manuscript; TM, KB, and MM provided experimental resources; GR, KB, and TM critically reviewed the manuscript. All authors read and approved the final manuscript.

## Funding

This research was financially supported by Elanco Animal Health and the Agriculture Innovation Program of Agriculture and Agri-Food Canada.

### Conflict of interest statement

The authors declare that the research was conducted in the absence of any commercial or financial relationships that could be construed as a potential conflict of interest. The reviewer AB and handling Editor declared their shared affiliation, and the handling Editor states that the process nevertheless met the standards of a fair and objective review.
